# Editorial: In celebration of women in developmental epigenetics

**DOI:** 10.3389/fcell.2024.1416081

**Published:** 2024-05-27

**Authors:** Mellissa R. W. Mann, Masako Suzuki, Claudia I. Keller Valsecchi

**Affiliations:** ^1^ Magee-Womens Research Institute, Department of Obstetrics, Gynecology and Reproductive Sciences, University of Pittsburgh School of Medicine, Pittsburgh, PA, United States; ^2^ Department of Nutrition, Texas A&M University, College Station, TX, United States; ^3^ Institute of Molecular Biology, Mainz, Germany

**Keywords:** epigenetics, development, women researchers, genomic imprinting, epigenetic programming, developmental exposures

According to the United Nations, only 33% of researchers worldwide are women. This number drops dramatically as women move through the academic ranks. Each year, more women professors leave academia (6% assistant; 10% associate; 19% full, 2011–2020, United States), and fewer women are promoted (associate, 7%; full, 12%) compared to men (Spoon et al., 2023). Similarly, in Europe, retention rates in STEM remain low, with only 19% of women at the senior level (=full professor, 2021). These figures are reflected in manuscript submissions, where only 4%–22% of corresponding authors are women (Nature Editorial, 2024; Brück, 2023; Cell Editorial Team, 2022). Further compounding the gender disparity in publishing, women at all levels (graduate students to faculty) are less likely to be credited with authorship than men (Ross et al., 2022). To counteract these trends, this Research Topic is dedicated to publishing manuscripts by women scientists as the first and/or corresponding authors.

Women scientists have made pioneering contributions to the field of epigenetics. Mary Lyon’s discovery of *X*-chromosome inactivation provided fundamental insights into dosage compensation mechanisms in mammals. Nobel laureate Barbara McClintock’s work, on transposons and epigenetic silencing challenged traditional genetic paradigms and emphasized dynamic gene regulation. Susan Clark’s development of bisulfite mutagenesis techniques was the bedrock for the precision mapping of global DNA methylation. Sarah Elgin’s pioneering research on heterochromatin structure and function in *Drosophila* was key to understanding position effect variegation. These women, and many others, stand as role models for women scientists. Here, we highlight the contributions of these articles as a celebration of women in developmental epigenetics.

## Genomic imprinting

Genomic imprinting is an epigenetic process that is dependent on the sex of the parent in which one parental allele is silenced, while the other parental copy is expressed (Barlow and Bartolomei, 2014). Genomic imprinting and its intersection with development have long been championed by women researchers, such as Denise Barlow, Marisa Bartolomei, Shirley Tilghman, and Anne Ferguson-Smith, who identified the first imprinted genes (Barlow et al., 1991; Bartolomei et al., 1991; Ferguson-Smith et al., 1991). In this Research Topic, Weinberg-Shukron et al. reviewed the developmental regulation of the *Dlk1*-*Dio3* imprinted domain. The authors conclude with a discussion on “how to build an imprinted domain.” Fang and colleagues also reviewed mechanisms of imprint regulation, assessing evidence for host defense mechanisms and endogenous retroviral elements in the establishment and maintenance of canonical and non-canonical imprints. Regmi et al. discovered that the *Dnmt1* P allele mutation reduced methylation levels throughout the mouse genome, except at gametic differentially methylated regions (DMRs). This protection did not extend to the corresponding secondary DMRs, suggesting that the maintenance mechanisms at gDMRs are different from those at non-imprinted sequences and secondary DMRs.

## Epigenetic programming

Given the reliance of epigenetic modifications on metabolites (e.g., methyl groups), investigators have turned to analyses of the one-carbon cycle and nutrients to decipher their role in embryonic/fetal epigenetic programming and inheritance (Clare et al., 2019). Emma Whitelaw’s pioneering work on the molecular regulation of the mouse Agouti locus exemplifies the interplay between nutrient sensitivity, environmental factors, transposable elements, and epigenetic regulation during development (Morgan et al., 1999). She coined the term metastable epialleles (MEs) to describe loci where DNA methylation status, and thus phenotype, varies between individuals (Rakyan et al., 2002). In this Research Topic, Sainty et al. reviewed the current knowledge on the early life environment, including maternal micronutrient availability, and disease risk later in life, with a specific focus on DNA methylation at MEs. Additionally, the authors describe the uniqueness of assessing DNA methylation in the placenta as a target tissue for studying MEs in mixed environmental exposures. Senner and co-authors investigated genome-wide DNA methylation in the placentas of mice with fetal growth restriction, using a hypomorphic mutation at the methionine synthase reductase gene, which encodes a key enzyme in one-carbon metabolism. Although regions with altered DNA methylation were identified in homozygous mutant placentas, including young endogenous retroviral elements with ectopic expression, a direct link between the methylome of mutant spermatozoa and that of mutant placentas was not found. Thus, the authors discounted DNA methylation as a mechanism for direct or multigenerational epigenetic inheritance of aberrant fetal growth. Ducreux and colleagues investigated the impact of commercial media and methionine supplementation on the embryonic transcriptome as a proxy for preimplantation epigenetic programming in human ART-produced embryos. Embryos cultured in Fericult (no amino acids) until day 2 had altered gene expression compared to those cultured in a Global medium, including downregulation of SETDB1, a lysine methyltransferase (H3K9me3). Further culture in Global until day 5 (Fericult-Global vs. Global-Global) minimized these transcriptional changes.

Epigenetic modifications are not limited to chromatin modifications. Non-coding RNAs (ncRNAs) also play a significant role in epigenetic regulation, including microRNAs, SINEUPs (natural antisense long ncRNAs that increase translation of partially overlapping mRNAs), telomerase RNAs, and promoter-associated long ncRNAs (Mattick and Makunin, 2006; Esteller, 2011). These ncRNAs interact with RNA-binding proteins to regulate gene expression, chromatin structure, and telomere length (Statello et al., 2020). In this Research Topic, Tokunaga and Imamura discussed the potential of analyzing ncRNAs to provide a new therapeutic approach to microcephaly, which is often associated with developmental disorders. Additionally, Le Breton et al. summarized the current understanding of the role of transposable elements (TEs) in the aging brain and in neurological conditions. They encouraged the investigation of aberrant TE activities and resulting products as potential biomarkers for neurological disorders or biological age.

## Developmental exposures

Given the malleability of epigenetic modifications to cellular signals, it is not surprising that they also respond to environmental exposures (Ryznar et al., 2021; Mo et al., 2022; Tando and Matsui, 2023). This is especially true during prenatal and perinatal development, with epigenetic perturbation contributing to long-term adverse health outcomes. Using *C. elegans* as a model system, Susan Gasser examined chromatin organization and Histone 3 lysine 9 methylation in relation to perinuclear anchoring to the nuclear scaffold, as well as changes in chromatin state, phenotypic plasticity, and developmental fate in response to environmental factors, such as overcrowding pheromones (Meister et al., 2011; Gonzalez-Sandoval et al., 2015). Lawless and colleagues reviewed the impact of prenatal cadmium exposure on epigenetic alterations in the placenta, fetus, child/offspring, and adult, including in germ cells, potentially contributing to adverse multigenerational effects. Petroff and co-authors examined the effects of environmental toxicants on hydroxymethylation. Exposure to the plasticizer di (2-ethylhexyl) phthalate over a period from preconception to perinatal weaning in mice resulted in aberrant hydroxymethylation in male (blood and cortex) and female (blood) adults. Similar exposure to lead (Pb) altered hydroxymethylation only in the cortex of adult males. These findings emphasize the susceptibility of the developing male cortex to environmental toxicants.

## Conclusion

This Research Topic serves as a flagship for all current and future women scientists and leads by example in making progress toward gender parity in publishing.

## References

[B1] Barlow D. P. Bartolomei M. S. (2014). Genomic imprinting in mammals. Cold Spring Harb. Perspect. Biol. 6 (2), a018382. 10.1101/cshperspect.a018382 24492710 PMC3941233

[B2] Barlow D. P. Stöger R. Herrmann B. G. Saito K. Schweifer N. (1991). The mouse insulin-like growth factor type-2 receptor is imprinted and closely linked to the TME locus. Nature 349 (6304), 84–87. 10.1038/349084a0 1845916

[B3] Bartolomei M. S. Zemel S. Tilghman S. M. (1991). Parental imprinting of the mouse H19 gene. Nature 351 (6322), 153–155. 10.1038/351153a0 1709450

[B4] Brück O. (2023) “A bibliometric analysis of the gender gap in the authorship of leading medical journals.” Commun. Med. 3, no. 179. 10.1038/s43856-023-00417-3 38082119 PMC10713825

[B5] Cell Editorial Team (2022). Assessing gender disparity among cell authors. Cell. 185 (5), 747–749. 10.1016/j.cell.2022.02.001 35150613

[B6] Clare C. E. Brassington A. H. Kwong W. Y. Sinclair K. D. (2019). One-carbon metabolism: linking nutritional biochemistry to epigenetic programming of long-term development. Annu. Rev. Animal Biosci. 7 (1), 263–287. 10.1146/annurev-animal-020518-115206 30412672

[B7] Editorial (2024). Nature publishes too few papers from women researchers - that must change. Nat. News. 10.1038/d41586-024-00640-5 38448701

[B8] Esteller M. (2011). Non-coding RNAS in human disease. Nat. Rev. Genet. 12, 861–874. 10.1038/nrg3074 22094949

[B9] Gonzalez-Sandoval A. Towbin B. D. Kalck V. Cabianca D. S. Gaidatzis D. Hauer M. H. (2015). Perinuclear anchoring of H3K9-methylated chromatin stabilizes induced cell fate in C. Elegans embryos. Cell. 163 (6), 1333–1347. 10.1016/j.cell.2015.10.066 26607792

[B10] Mattick J. S. Makunin I. V. (2006). Non-coding RNA. Hum. Mol. Genet. 15 (Suppl. l_1), R17–R29. 10.1093/hmg/ddl046 16651366

[B11] Meister P. Mango S. E. Gasser S. M. (2011). Locking the genome: nuclear organization and cell fate. Curr. Opin. Genet. Dev. 21 (2), 167–174. 10.1016/j.gde.2011.01.023 21345665 PMC4041333

[B12] Mo J. Liu X. Huang Y. He R. Zhang Yu Huang H. (2022)“Developmental origins of adult diseases.” Med. Rev. 2: 450–470. 10.1515/mr-2022-0027 PMC10388800 37724166

[B13] Morgan H. D. Sutherland H. G. E. Martin D. I. K. Emma W. (1999). Epigenetic inheritance at the Agouti locus in the mouse. Nat. Genet. 23 (3), 314–318. 10.1038/15490 10545949

[B14] Rakyan V. K. Blewitt M. E. Druker R. Preis J. I. Whitelaw E. (2002). Metastable epialleles in mammals. Trends Genet. 18 (7), 348–351. 10.1016/s0168-9525(02)02709-9 12127774

[B15] Ross M. B. Glennon B. M. Murciano-Goroff R. Berkes E. G. Weinberg B. A. Lane J. I. (2022). Women are credited less in science than men. Nature 608 (7921), 135–145. 10.1038/s41586-022-04966-w 35732238 PMC9352587

[B16] Ryznar R. J. Phibbs L. LonVan J. W. (2021). Epigenetic modifications at the center of the barker hypothesis and their transgenerational implications. Int. J. Environ. Res. Public Health 18, 12728. 10.3390/ijerph182312728 34886453 PMC8656758

[B17] Spoon K. LaBerge N. Hunter Wapman K. Zhang S. Morgan A. C. Galesic M. (2023). Gender and retention patterns among U.S. Faculty. Sci. Adv. 9 (42), eadi2205. 10.1126/sciadv.adi2205 37862417 PMC10588949

[B18] Statello L. Guo C.-J. Chen L.-L. Huarte M. (2020). Gene regulation by long non-coding RNAS and its biological functions. Nat. Rev. Mol. Cell. Biol. 22 (2), 96–118. 10.1038/s41580-020-00315-9 33353982 PMC7754182

[B19] Tando Y. Matsui Y. (2023). Inheritance of environment-induced phenotypic changes through epigenetic mechanisms. Environ. Epigenetics 9 (1), dvad008. 10.1093/eep/dvad008 PMC10719065 38094661

